# Thyroid testing paradigm switch from thyrotropin to thyroid hormones—Future directions and opportunities in clinical medicine and research

**DOI:** 10.1007/s12020-021-02851-6

**Published:** 2021-08-27

**Authors:** Stephen P. Fitzgerald, Nigel G. Bean, James V. Hennessey, Henrik Falhammar

**Affiliations:** 1 The Departments of General Medicine and Endocrinology, The Royal Adelaide Hospital, Adelaide, SA South Australia; 2 The University of Adelaide, School of Medicine, Adelaide, SA Australia; 3 School of Mathematical Sciences and ARC Centre of Excellence for Mathematical and Statistical Frontiers, University of Adelaide, Adelaide, SA Australia; 4 Division of Endocrinology, Department of Medicine Beth Israel Deaconess Medical Center, Harvard Medical School, Boston, MA USA; 5 Department of Endocrinology, Karolinska University Hospital, Stockholm, Sweden; 6 Department of Molecular Medicine and Surgery, Karolinska Institutet, Stockholm, Sweden; 7 Menzies School of Health Research and Royal Darwin Hospital, DARWIN, NT Australia

**Keywords:** Thyroid state, Thyroid function, Thyrotropin (TSH), Thyroid hormones

## Abstract

**Purpose:**

Recently published papers have demonstrated that particularly in untreated individuals, clinical parameters more often associate with thyroid hormone, particularly free thyroxine (FT4), levels than with thyrotropin (TSH) levels. Clinical and research assessments of the thyroid state of peripheral tissues would therefore be more precise if they were based on FT4 levels rather than on TSH levels. In this paper we describe implications of, and opportunities provided by, this discovery.

**Conclusions:**

The FT4 level may be the best single test of thyroid function. The addition of free triiodothyronine (FT3) and TSH levels would further enhance test sensitivity and distinguish primary from secondary thyroid dysfunction respectively. There are opportunities to reconsider testing algorithms. Additional potential thyroidology research subjects include the peripheral differences between circulating FT4 and FT3 action, and outcomes in patients on thyroid replacement therapy in terms of thyroid hormone levels. Previously performed negative studies of therapy for subclinical thyroid dysfunction could be repeated using thyroid hormone levels rather than TSH levels for subject selection and the monitoring of treatment. Studies of outcomes in older individuals with treatment of high normal FT4 levels, and pregnant women with borderline high or low FT4 levels would appear to be the most likely to show positive results. There are fresh indications to critically re-analyse the physiological rationale for the current preference for TSH levels in the assessment of the thyroid state of the peripheral tissues. There may be opportunities to apply these research principles to analogous parameters in other endocrine systems.

In the absence of other identified reliable clinical markers [1] it has been accepted practice over recent decades to base the initial assessment of the thyroid state of peripheral tissues on circulating levels of thyroid stimulating hormone (TSH) [2]. This has been an exceptional practice as generally in medicine and physiology the level of the relevant parameter is used to determine the presence of normality/abnormality and the level of the controlling hormone is used to determine the cause of any abnormality [3].

Recent work, however, has indicated that analogously to the situation in other systems, clinical parameters, particularly in patients not on thyroid treatments, are associated more often with circulating thyroid hormone levels and especially free thyroxine (FT4) levels than with TSH levels [4, 5]. In fact, in our meta-analysis [4] none of the examined clinical parameters associated more often with TSH levels than with thyroid hormone levels.

As the thyroid state of peripheral tissues and of the individual as a whole is an integration of multiple clinical parameters, the above work therefore provides strong evidence that the definitions of euthyroidism, borderline thyroid dysfunction, subclinical thyroid dysfunction and overt thyroid dysfunction, in terms of the peripheral thyroid state, are better defined by thyroid hormone levels than TSH levels.

In this paper we propose some of the implications and opportunities presented by this evidence supporting a switch from TSH levels to thyroid hormone levels in the biochemical assessment of the peripheral thyroid state.

A change in emphasis to the use of thyroid hormone levels would affect the interpretation of thyroid function tests. We propose that biochemical euthyroidism of peripheral tissues be indicated by normal levels of thyroid hormones, particularly levels of FT4, regardless of the TSH level, and that borderline thyroid dysfunction be indicated by levels of thyroid hormones adjacent the limits of the population normal range. Consistent with general assessment methodology [3], TSH levels would still remain valuable in defining the relative contributions of the thyroid gland and the hypothalamus-pituitary to any given thyroid state. TSH levels may also be relevant in the circumstances of there being direct peripheral effects of TSH [6].

Though subclinical thyroid dysfunction as defined using TSH levels is associated with clinical parameters the evidence suggests that these associations are indirect, a consequence of the strong population correlation of FT4 with TSH. Nevertheless, the concept of subclinical thyroid dysfunction retains utility. We propose that rather than being continued to be considered a precise indicator of the peripheral thyroid state, subclinical thyroid dysfunction be regarded as an indicator of thyroid gland compromise. As dysthyroidism is usually a result of primary thyroid gland dysfunction, the continuing validity of subclinical thyroid dysfunction as a risk factor for the development of overt thyroid dysfunction [7] is coherent with physiological principles.

Such changes would be expected to affect testing strategy. No single test of thyroid function is infallible; ideally clinical assessment would be combined with levels of circulating FT4, free triiodothyronine (FT3) and TSH. Our meta-analysis [4] suggests that should a single assay be used for screening the FT4 level may give the most precise guide to the thyroid state of peripheral tissues. Adding a FT3 level would remove the possibility of missing the uncommon entity of T3 toxicosis. TSH levels alone, as well as being unreliable in the context of central disease, are less precise than FT4 levels in determining borderline states [4].

The omission of measuring TSH levels in initial testing would almost certainly lead to a reduction in the frequency of the diagnosis of subclinical thyroid dysfunction. There are opportunities to examine the consequences of this, as well as to re-examine the cost-benefits of different testing strategies. The use of thyroid hormone levels for the assessment of the peripheral thyroid state provides the additional benefit of being cognitively simple and natural. The use of TSH levels can be confusing (particularly for patients) in that they are an inverse measure of the thyroid state.

Clinical guidelines, particularly those devoted to the diagnosis and treatment of hypothyroidism [8] and thyroid disorders of pregnancy [9] contain large sections based on TSH-level based diagnosis and management. Opportunities exist to re-formulate these guidelines, particularly in terms of diagnosis.

As the studies of the relationship between clinical states and hormone levels have included patients predominantly not taking thyroid hormones there are opportunities to repeat the studies specifically examining subjects on different replacement regimes. Thus, the validity of the seemingly logical extrapolation of the above principles to these groups of patients in terms of management and monitoring the thyroid state might be tested. It seems that for pharmacokinetic and other reasons, different levels of thyroid hormones (and possibly TSH levels) may be optimal in different circumstances of thyroid replacement [8, 10].

The use of thyroid hormone levels to assess the peripheral thyroid state may be a secondary consideration in the monitoring of thyroid replacement therapy in the context of thyroid cancer if suppression of TSH levels is the primary goal [11]. Furthermore, this paper concerns assessment of the thyroid state in the general population. In circumstances of disturbed thyroid hormone action or regulation whether due to genetic factors, illness or drugs, the assessment of the peripheral thyroid state becomes more complex.

Further research on the relative importance of the different thyroid hormones may also be valuable. Though in our meta-analysis [4] FT3 associated with clinical parameters at least as often as did FT4, more sophisticated analyses suggested that FT4 levels may be the most robust measurement of the thyroid state in general. In particular many of the associations with FT3 may have been the result of reverse-causation. In non- thyroidal illness, including for example cancer, low FT3 levels may be a consequence of the clinical state [12] thereby being associated with adverse prognoses [13]. This is to be distinguished from for example high normal FT4 levels increasing the risk of atrial fibrillation. Nevertheless, there is evidence that different tissues may respond differently to FT4 and FT3 [14]. Opportunities exist to investigate the underlying physiological bases of any such differences. We anticipate that such research would include the roles of circulating FT4 and FT3, and tissue deiodinases, in intracellular T3 (the active hormone) homeostasis.

It remains possible that in some circumstances TSH levels are superior to thyroid hormone levels as a measure of the peripheral thyroid state. Thus far, however, this has not applied to any of the parameters we and others have examined [4, 5]. Other authors have suggested that combining the thyroid hormone levels with TSH levels may best indicate the peripheral state [15]. These hypotheses could be tested.

The thyroid state is significantly associated with clinical outcomes across a wide range of peripheral systems. These outcomes include cardiovascular pathologies including atrial fibrillation [16], risks of dementia [17], death [18], bone loss [19], metabolic syndromes [20] and pregnancy complications [21–23]. The variations in outcomes may be significant, i.e. comparable to those seen with conventional risk factors, even with variations of thyroid function within the normal range. Thyroid hormone levels too therefore might be regarded as risk factors in the same way we regard the risk factor blood pressure, with the relevant risks being continuous across the range rather than being restricted to abnormal states.

Thus far, however, studies of therapeutic interventions have generally been negative [24, 25]. We would argue that these studies may have been flawed in that the basis for the identification of subjects has been on the basis of TSH levels and in particular on the use of TSH levels as a definition of subclinical thyroid dysfunction. A change in the paradigm to the use of FT4 levels provides the opportunity to re-evaluate the potential benefits of treating borderline thyroid function with more precise selection of suitable subjects.

In addition, because primary changes to FT4 levels result in larger changes to TSH levels, previous trials of interventions in subclinical thyroid dysfunction explored the effects of small changes, e.g. ~2 pmol/L [24], in FT4 levels (these small changes being sufficient to normalise TSH levels). The use of FT4 levels for the determination of the peripheral thyroid state in trials would not only allow more precise selection of appropriate subjects but would also allow for more vigorous titration of intervention changes, even to the point of TSH levels becoming abnormal.

The strongest associations of thyroid hormone levels and clinical parameters may be the associations of thyroid hormone levels in the upper part of the normal range with atrial fibrillation [16], dementia [17] and death [18]. It appears that higher levels of thyroid hormones may become a significant risk factor with aging. There is sufficient rationale to conduct a prospective study of the above and other outcomes in older individuals with and without an intervention to lower thyroid hormone levels, using thyroid hormone levels and in particular FT4 levels as the relevant variable. There might be separate study/subgroup analysis of individuals taking thyroid replacement hormones.

Despite concerns that FT4 assays may be flawed during pregnancy [26], in our meta-analysis [4] pregnancy outcomes were more often associated with FT4 levels than with TSH levels. Therefore, studies of pregnancy outcomes using thyroid hormone replacement aiming for mid-range levels of FT4 also appear to be justified and would have the advantage of needing relatively short observation time spans.

There are now opportunities to critically reconsider the previous theoretical rationale [2], supporting the current practice. This rationale has been largely based on the concepts of a thyroid ‘set point’ and ‘individual euthyroidism’ [2] as well as the ‘precision’ of TSH levels in the determination of the thyroid state [2] and concerns re assay accuracy [26]. We have already provided evidence that denies the validity of thyroid set points and individual euthyroidism [27]. Further re-analysis of these and other related physiological concepts [10] are likely to provide a physiological model more coherent with the available evidence.

The principles of our work might also be extended to parameters other than thyroid function. The range of normal levels of other hormones may also have associations with clinical parameters [28, 29] and in some individuals, particularly those whose levels are at the edge of the normal range might benefit from intervention. There may be individuals with multiple different hormone levels that are normal but all contributing to risk. The combination of high normal thyroid hormones and high normal estrogen levels, each individually associated with breast cancer [28, 30], might for example be a treatment target.

In summary the recent developments in the study of fundamental aspects of thyroid hormone regulation and action present opportunities to refine clinical care and to pursue new directions in research in thyroidology and in general endocrinology.
